# Socioeconomic Inequality in Mortality from Road Traffic Accident in Iran

**Published:** 2019-01-01

**Authors:** Fatemeh Shahbazi, Seyed Saeed Hashemi Nazari, Hamid Soori, Soheila Khodakarim

**Affiliations:** ^1^ Department of Epidemiology, School of Public Health and Safety, Shahid Beheshti University of Medical Sciences, Tehran, Iran; ^2^ Safety Promotion and Injury Prevention Research Center, School of Public Health and Safety, Shahid Beheshti University of Medical Sciences, Tehran, Iran; ^3^ Department of Epidemiology, School of Paramedical Science, School of Public Health and Safety, Shahid Beheshti University of Medical Sciences, Tehran, Iran

**Keywords:** Road traffic accident, Inequality, Mortality, Injury, Concentration index, Socioeconomic status

## Abstract

**Background:** Although the epidemiology of road traffic accidents (RTAs) and their determining factors have been extensively investigated and debated in Iran, yet there is no data regarding socioeconomic inequalities in mortality from RTA in Iranian context. Since effective interventions to control, management and diminish the negative influence of RTAs would require understanding of numerous contributing factors, including the inequalities We aimed to quantify for the first time the socioeconomic differential in mortality or injuries from RTAs.

**Study design:** A cross-sectional study.

**Methods:** Overall, 50755 died and injured people from RTA from Mar 2015 to Feb 2016 were evaluated. The data were taken from traffic police department in Iran. We calculated concentration index (CI) to measure socioeconomic inequality in traffic-related mortality & injury. Data were analyzed using Stata software.

**Results:** The mortality and injury rate from accidents was 1130.80 per 10000 accidents. The concentration index was negative for mortality rate (-0.11) of RTA, indicating the higher concentration of the rates among deprived groups.

**Conclusion:** People with low socioeconomic level were more at risk for fatal accidents and injuries. Therefore, specific interventions on road safety should be delivered in the deprived drivers which uses from low safety level vehicles.

## Introduction

Road traffic accidents (RTAs) have become a dangerous public health problem all over the world^1^. This issue is a leading cause of mortality, morbidity, and disability, especially in low and middle-income countries^2-4^. Every year between 20-50 million peoples suffer from non-fatal injuries and many of them acquire a disability as a consequence of their injury and the lives of almost 1.25 million men and women are cut concise as a result of RTAs^5^. A significant proportion of these deaths occur in south-western Asia and Iran is located in this area^6^.

RTAs have been reported as the second leading cause of death and the first leading source of years of life lost in Iran^7,8^. In this realm, inside city and outside city crashes have become a substantial threat to health that making Iran one of the highest ranking countries in terms of vehicular injuries and RTAs^9^.

The effective interventions to control, management and diminish the negative influence of RTAs would require understanding of numerous contributing factors, including the socioeconomic inequalities,^10^, not well studied in Iranian literature.

Numerous studies have demonstrated the socioeconomic inequalities in traffic accidents and fatalities^11-14^. The study of socioeconomic inequalities in health branches such as traffic injuries is equity purposes and social justice, since targeting injury preventive measures toward disadvantaged groups may increase the overall effectiveness of these measures^15^.

Although the epidemiology of RTAs and their determining factors have been extensively investigated and debated in Iran ^7,8, 16-18^; yet there is no data regarding socioeconomic inequalities of these adverse events in Iranian context. Consequently, we aimed to show the socioeconomic differences in mortality or injuries from RTAs. This research will contribute to a deeper understanding of epidemiology and inequalities related to RTA and provide essential information for planning and also further researches.

## Methods

### Study population

The study was conducted using data on accidents that had led to injury or death or damage to the car were extracted from traffic police department (TPD) during 12 month periods (Mar 2015-Feb 2016). TPD records RTA deaths that occur in the accident scene. In this database all the crashes which death or injury happens to the passengers of a vehicle is damaged, are registered. The accidents without any injury or property damage are not registered.

### Outcome Measurement

We considered all accidents which at least one car was involved in their occurrence. The accidents caused by heavy vehicles (for example bus, minibus, truck, lorry and so on); were excluded. If the driver or one of the passengers of a car were injured in an accident, that car was considered as outcome positive, otherwise, it was considered as outcome negative.

### Socioeconomic Status

The price of the cars was used for socioeconomic status classification. On this basis, each car was allocated to one of the following socioeconomic groups including the poorest (under 2500 $), poor (2500-5000 $), average (5000-7500 $), rich (7500 -10000 $) and the richest (higher than 10000 $).

In the second analysis for further understanding the observed inequality, we limited our analysis to just the most common car brands in Iran which are Pride, Peykan, Samand, Tiba, Peugeot and Tondar 90). These cars had the largest proportion in the incidence of the inside city and outside city accidents.

These cars were ordered according to their price from the lowest to highest as follows: Peykan, Pride & Tiba, Samand, Peugeot and Tondar 90; which represents an increase in the economic class of the car; or, in other words, their arrangement demonstrates the improvement of the socioeconomic situation.

### Statistical analysis

RTA mortality and injury rates were estimated by dividing the number of RTA deaths or injuries in every car economic category by the total numeral of crashes in each group. The rates are presented per 10,000 accident involving cars.

### Inequality measure

Inequality is calculated by measuring the concentration index (CI). CI is one of the best measures of inequalities in social group with a natural ordering. Most researchers have used this index to measure inequality^19^. CI is calculated based on the concentration curve (CC), where plot the cumulative percentage of mortality or injury from accident (y-axis) against the cumulative percentage of cars ranked by their socioeconomic status (x-axis) beginning with the poorest SES (left), and ending with the richest SES (right). The CI is defined as twice the area between the CC and line of inequality (diagonal). The value of CI can vary between -1 and +1. When CC tangent with the diagonal, the CI is equal to zero usually indicating no inequality in mortality & injury from RTA; when CC lie above the diagonal the CI is negative and indicating the outcome is more concentrated among cars with lower SES and vice versa when CC lie below the diagonal, the CI is positive and indicating the outcome is more concentrated among cars with higher SES. The close-up of CI to -1 and +1 represent more inequality. The Stata commands including; Igini and Clorenz were used to calculate of CI and CC respectively. We processed and analyzed data using the Stata software version 14 and DASP statistical package version 14. Data were analyzed at 0.05% significance level.

## Results

During the study period, 366,767 accidents resulting in injury or damage had been registered which 610643 vehicles were involved in these accidents. After excluding heavy cars and also motorcycles, 453,014 light cars were involved in the accidents which the type of accident in 42,015 cases was injury/death, in 400,923 (88.5%) cases were just physical damage and in the remainder (0.02%) were unknown. The mortality or injury rate from light cars (per 10,000 accidents) was 927.45.

**Figure 1:**
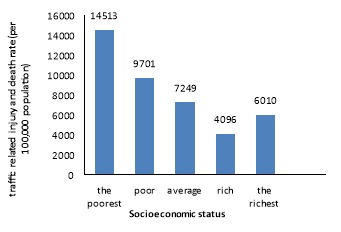
Mortality/injury rate from road traffic accidents in 12 months leading to Feb 2016 in Iran

**Figure 2:**
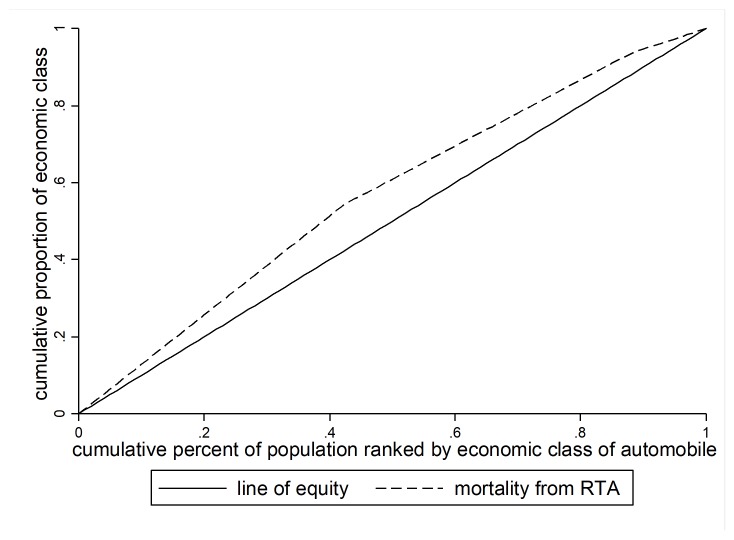
Concentration curve for all socioeconomic category of light cars

The mortality and injury rate from accidents was 1130.80 per 10,000 accidents. Figure 1 displays the mortality/injury rate from road traffic accidents in each socioeconomic status. An apparent negative relationship demonstrates between socioeconomic level and mortality or injury rate from RTA: the lower the socioeconomic status, the higher the mortality or injury rate. This trend was further confirmed by the concentration curve, which lies above the line of equality (Figure 2 ). Negative value of concentration index (concentration Index: -0.11 & 95 % CI: -0.24; -0.04) and result from Figure 2 indicate that death or injury rates of RTA is more concentrated in low socioeconomic groups; in other word deaths and injuries have been concentrated in crashes involving low price cars.

**Figure 3:**
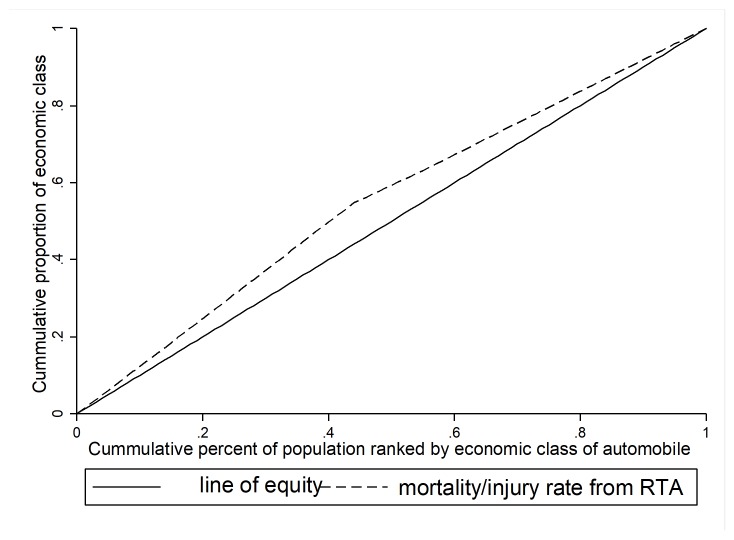
Concentration curve for the most common cars in Iran

Inequality in injury/death rate among 5 most common cars in Iran:

After observing the inequality in the death/injury rate from RTA between depriving socioeconomic classes; in order to better understand this disparity, we chose the automobiles that had the most proportion in the poor and poorest SES groups; subsequently, we calculated the inequality in mortality or injury rate from RTA among these vehicles. In this situation, the number and rates of mortality/injury from RTAs by Iranian common cars in 12 months leading to Feb 2016 in Iran is more concentrated in low socioeconomic groups (concentration index: -0.10 and 95% CI:-0.12; -0.09). Generally, 72.23% of deaths and injuries from light cars occur in common cars (Table 1) (Figure 3).

**Table 1:** Crude death/ injury counts and rates for road traffic accidents by common cars in 12 months leading to Feb 2016 in Iran

**Type of automobile**	**Number of crashes**	**Rate per 10000 cars involving accidents**
Peykan	6538	1503.40
Pride & Tiba	15464	1509.71
Samand	3190	943.95
Peugeot	14076	1002.85
Tondar 90	399	922.97

The overall concentration index for death/injury rate from RTA between the most common cars was -0.10 (95% confidence interval -0.12, -0.09). Thus death/injury rate from crashes among the common automobiles was concentrated more at the poorer end of the price distribution of cars.

## Discussion

This study showed that the inequality existed in mortality and injury rates from RTAs. The negative value of concentration index in deaths and injuries from traffic accident (-0.11 & -0.10) indicate that population with disadvantaged SES in Iran are more likely to suffer from adverse consequences of traffic accidents, which is consistent with previous studies in other countries ^1,10,12,20-23^.

Based on our results, the deaths and injuries from traffic crashes have been concentrated among people who are using low priced cars. These vehicles do not have advanced safety features (such as airbag, ABS brakes, etc.). Crash test rating of these cars is also lower^24^. Improvements in vehicle safety have played an important role in reducing RTA death rates. To the extent that the distribution of safer vehicles between lower socioeconomic groups delays because of higher costs or other mechanisms, we will encounter with higher inequalities in RTAs among socioeconomic groups of the society^25^. Low educational level, behavioral risk factors such as alcohol consumption during driving, lower adherence to seat belt use and contextual factors are risk factors for driving accidents which are more concentrated among people who have low SES^26-31^.

The current study had some limitations. First of all, the cross-sectional nature of the survey data would have little implication of causality. Second, we used the price of the car as a measure of SES, but alternative indicators (such as education or income) could show different results. Third, we used TPD as a source of death & injury registration thus, an underestimation of the magnitude of RTA mortality or laceration might have occurred via under-reporting of death (the death that occurs on the way to the hospital or in the hospital are not registered in police reports). Forth, regarding the fact that traffic police department registers the deaths that occur in the accident scene and many of the injured people die on the way to hospital or in the hospital, we did not report the mortality and injury rates separately.

## Conclusion

Poor and poorer drivers bear a significantly heavier burden of accident-related injury and mortality in Iran, which require considerate commitments and resources from Iranian government and other stakeholders to eliminate the socioeconomic inequality in RTAs. When planning programs for injury prevention, effective interventions should focus on the groups in greatest need, without widening the gap between socioeconomic differences in health outcomes. Additional work should investigate potential risk factors and sources of inequality by decomposition methods.

## Acknowledgements

We are grateful to Department of Traffic Police for providing the data of this research. This study is part of an MSc thesis of Epidemiology in the Shahid Beheshti University of Medical Sciences, Iran.

## Conflict of interest statement

The authors declare that there is no conflict of interests.

## Funding

This study is part of an MSc thesis of epidemiology in the Shahid Beheshti University of Medical Sciences, Iran. It is financially supported by the Shahid Beheshti University of Medical Sciences and also Legal Medicine Organization of Iran.

## Highlights

- One of the important goals of any health care system is to assess and reduce inequalities in health outcomes.
- Based on the previous research there is SES inequalities in mortality from RTA.
- The mortality and injury rate from accidents was 1130.80 per 10000 accidents.
- Mortality from RTAs is concentrated among people with low socioeconomic status.
- Mortality from RTAs is concentrated among people which use automobiles with low safety level.
